# 80 Validation of PROMIS-25 Among Children Living with Burn Injuries

**DOI:** 10.1093/jbcr/irac012.083

**Published:** 2022-03-23

**Authors:** Kara McMullen, Alyssa M Bamer, Andrew Humbert, Colleen M Ryan, Jeffrey C Schneider, Lewis E Kazis, Barclay T Stewart, Oscar E Suman-Vejas, Dagmar Amtmann

**Affiliations:** University of Washington, Portland, Oregon; University of Washington, Golden, Colorado; University of Washington, Seattle, Washington; Harvard Medical School, Boston, Massachusetts; , Massachusetts; Boston University School of Public Health, Spaulding Rehabilitation Hospital, Harvard Medical School, Boston, Massachusetts; University of Washington, Seattle, Washington; U of Texas Medical Branch, Galveston, Texas; University of Washington, Seattle, Washington

## Abstract

**Introduction:**

Patient-reported outcomes are important for burn injury research and clinical practice. The NIH-funded Patient Reported Outcomes Measurement System (PROMIS)-25 profile has been validated for use in diverse populations of children with many conditions, though not among children living with burn injuries. The purpose of this study was to examine the reliability and validity of PROMIS-25 scores in children living with burn injury.

**Methods:**

Data were provided by children who were participating in a multi-center longitudinal study of outcomes after burn injury. The PROMIS-25 Profile, which includes 4 items for each domain of physical function mobility, anxiety, depression, fatigue, peer relationships, and pain interference, was evaluated for reliability and validity. Floor and ceiling effects, unidimensionality, internal consistency, and reliability were examined. Correlations with other measures (Post-Traumatic Growth Inventory-Child (PTGI-C), Child PTSD Symptom Scale (CPSS) and Burn Outcome Questionnaire Body Image Scale (BOQBI)) were calculated to assess concurrent validity.

**Results:**

256 children living with burn injury who sustained a moderate to severe injury provided responses on PROMIS-25 domains 6 months-10 years post burn. Participants’ age ranged from 8-18 years at time of assessment; mean years since injury was 4.3 (SD 4.1). All PROMIS-25 domains showed high internal consistency (Cronbach’s *α*=0.90–0.95). Substantial portions of the sample reported no symptoms (anxiety [58.2%], depressive symptoms [54.6%], fatigue [50.8%], pain [60.1%]). There was a large ceiling effect on peer relationships (46.8%) and physical function mobility (57.5%). One-factor confirmatory factor analyses supported unidimensionality for all domains (all CFI >0.98). Reliability was credible for group mean comparisons ( >0.8) across at least some trait levels for all domains except fatigue and anxiety which had low reliability (< 0.8) across the entire trait range. The magnitude and direction of correlations were as anticipated (0.32 for peer relationships and body image; 0.51 for depressive symptoms and PTSD) with the exception of weak negative correlations between PTGI-C and the anxiety and depression domains.

**Conclusions:**

The results provide some evidence of reliability and validity of PROMIS-25 scores among children living with burn injury. Reliability of all domains was low to moderate and would likely be improved, and ceiling effects reduced, by administering the PROMIS-37, which includes 6 items per domain.

